# Supporting Women Undergoing IVF Treatment With Timely Patient Information Through an App: Randomized Controlled Trial

**DOI:** 10.2196/28104

**Published:** 2021-08-27

**Authors:** Thomas Timmers, Manouk Keijsers, Jan A M Kremer, Loes Janssen, Jesper Smeenk

**Affiliations:** 1 IQ healthcare Radboud Institute for Health Sciences Radboud University Medical Center Nijmegen Netherlands; 2 Interactive Studios Rosmalen Netherlands; 3 Department of Obstetrics and Gynaecology Elisabeth-TweeSteden Hospital Tilburg Netherlands; 4 Máxima Medical Center Veldhoven Netherlands

**Keywords:** patient education, fertilization in vitro, mobile health, health literacy, gynecology

## Abstract

**Background:**

Since the introduction of assisted reproductive technologies in 1978, over 2 million in vitro fertilization (IVF) babies have been born worldwide. Patients play a vital role in the success of this treatment. They are required to take fertility medication (hormone injections) to activate the ovaries to produce a sufficient number of oocytes. Later, they need to take medication to increase the chance of the embryo surviving inside the uterus. Patients are educated during an intake consultation at the start of the treatment to minimize the emotional burden and reduce noncompliance. The consultation lasts about 30 to 45 minutes and covers all essential subjects. Even though ample time and energy is spent on patient education, patients still feel anxious, unknowledgeable, and unsupported. As such, electronic health utilizing a smartphone or tablet app can offer additional support, as it allows health care professionals to provide their patients with the correct information at the right time by using push notifications.

**Objective:**

This randomized controlled trial aimed to evaluate the capacity of an app to support IVF patients throughout the different phases of their treatment and assess its effectiveness. The study's primary outcome was to determine the patients’ level of satisfaction with the information provided. The secondary outcomes included their level of knowledge, ability to administer the medication, overall experienced quality of the treatment, health care consumption, and app usage.

**Methods:**

This study was performed at a specialized fertility clinic of the nonacademic teaching hospital Elisabeth-TweeSteden Ziekenhuis in Tilburg, the Netherlands. Patients who were scheduled for IVF or intracytoplasmic sperm injection treatments between April 2018 and August 2019 were invited to participate in a physician-blinded, randomized controlled trial.

**Results:**

In total, 54 patients participated (intervention group: n=29). Patients in the intervention group demonstrated a higher level of satisfaction on a 0 to 10 scale (mean 8.43, SD 1.03 vs mean 7.70, SD 0.66; *P*=.004). In addition, they were more knowledgeable about the different elements of the treatment on a 7 to 35 scale (mean 27.29, SD 2.94 vs mean 23.05, SD 2.76; *P*<.001). However, the difference disappeared over time. There were no differences between the two patient groups on the other outcomes. In total, 25 patients in the intervention group used the app 1425 times, an average of 57 times per patient.

**Conclusions:**

Our study demonstrates that, in comparison with standard patient education, using an app to provide patients with timely information increases their level of satisfaction. Furthermore, using the app leads to a higher level of knowledge about the steps and procedures of IVF treatment. Finally, the app’s usage statistics demonstrate patients’ informational needs and their willingness to use an electronic health application as part of their treatment.

**Trial Registration:**

Netherlands Trial Register (NTR) 6959; https://www.trialregister.nl/trial/6959

## Introduction

### Background

Since the introduction of assisted reproductive technologies in 1978, over 2 million IVF (in vitro fertilization) babies have been born worldwide [1]. The technique offers infertile couples the chance to become pregnant and is currently applied over a million times annually in the United States and Europe [2-4]. An IVF or ICSI (intracytoplasmic sperm injection) treatment has many different stages and can easily take up to several months. First, there is the collection of oocytes (mature egg cells) from the ovaries that need to be fertilized by sperm in a lab. After successful fertilization, the oocytes are transferred to the uterus (embryo transfer). Pregnancy then depends, among different factors, on the embryos attaching to the lining of the uterus.

Patients’ behavior and adherence to treatment instructions play a vital role in the success of this treatment. First, they are required to take fertility medications (hormone injections) to activate the ovaries to produce a sufficient number of oocytes. Later, they need to take medication to increase the chances of the embryo surviving inside the uterus. The medication comes with very strict regimes in terms of application and timing, and most women suffer from the side effects of using the medications and experience stress related to the treatment process. Patients and clinicians report being anxious, which often results in nonadherence to the treatment process [5,6].

Patients are educated about the process during an intake consultation at the start of the treatment to minimize the emotional burden and reduce the risk of noncompliance. The consultation lasts about 30 to 45 minutes. It covers all the important subjects, including the physiology of the menstrual cycle, administration of the medication (and its side effects), oocyte retrieval and embryo transfers, risks, and the chances of becoming pregnant. Even though ample time and energy is spent on patient education, patients still feel anxious, unknowledgeable, and unsupported [5,7-13]. These emotions often relate to the feeling of being uninformed. In contrast, patients prefer being routinely provided with understandable, structured, and practical information regarding their IVF or ICSI treatments. [9,14-18]. Using eHealth via a smartphone or tablet app allows health care professionals to provide their patients with the right information at the right time through push notifications. These notifications may refer to newly available information, prepare patients for a consultation, or remind patients to take their medication and provide relevant instructions.

Furthermore, the information is readily available, complete, well-structured, and presented in different modes like text and video. It can utilize feedback systems to test and retest patients’ understanding of important information. A 2020 systematic review demonstrated the effectiveness of these interventions on many different outcomes, ranging from knowledge and satisfaction to adherence and quality of life [19].

### Objectives

This randomized controlled trial aimed to evaluate the capacity of an app to support IVF and ICSI patients throughout the different phases of their treatment and assess its effectiveness. The study's primary outcome was to determine the patients’ level of satisfaction with the information provided. The secondary outcomes included their level of knowledge, ability to administer the medication, the overall experienced quality of the treatment, and health care consumption. In addition, app usage statistics were gathered to assess the need for specific information in the app. We hypothesized that providing patients with timely information via an app would positively affect all outcomes compared to standard patient education practices.

## Methods

### Study Design

This study was performed at a specialized fertility clinic of the nonacademic teaching hospital Elisabeth-TweeSteden Ziekenhuis (ETZ) in Tilburg, the Netherlands. Patients who were scheduled for IVF or ICSI treatment were invited to participate in a physician-blinded, randomized controlled trial between April 2018 and August 2019. The study assessed the effectiveness of an interactive app in addition to the standard care (website and brochures) in a parallel-group design with an equal allocation ratio. The app was used to support and educate patients through the different stages of their treatment, ranging from the intake and medication instructions to the oocyte retrieval, embryo transfer, and pregnancy test. No changes were made to the study design after the study was initiated. We followed the CONSORT (Consolidated Standards of Reporting Trials) guidelines and the CONSORT eHealth checklist [20,21].

### Informed Consent and Ethical Considerations

The hospital staff asked patients to consider participating in the study following their first consultation with a fertility physician indicating they were eligible for IVF or ICSI treatment. Interested patients received all the necessary information about the study, and they were offered at least 2 days to reflect on the information. If they had any questions, they could contact the local research coordinator (MK, gynecology resident since 2018) by phone or email. If they agreed to participate in the study, patients signed the informed consent before initiating their treatment. The study was registered at the Netherlands Trial Registry (reference number 6959). The study was approved by the Institutional Review Board of the Maxima Medical Centre (Eindhoven, the Netherlands; reference number N18.030) and the ETZ hospital’s local review board.

### Participant Selection

Patients scheduled for IVF or ICSI treatments at the ETZ hospital were eligible for inclusion. Additionally, participants were required to be fluent in Dutch and possess an email address and a smartphone or tablet. For the remainder of this article, we will refer to this patient population (patients scheduled for IVF or ICSI treatment) as “IVF patients.”

### Intervention

The Patient Journey App (Interactive Studios) provided timely information to IVF patients in the intervention group. The app was only available to patients in the intervention group, and they obtained access to the app after completing the baseline questionnaire. They received an email with download instructions for the app and a personal code to enter on the app’s timeline to unlock the information in the app.

All patients in the intervention group received the same information via the app. However, the timing of the information and push notifications was based on the date a specific patient started the treatment and the date the patient underwent the oocyte retrieval. These dates were entered into the system by the hospital staff to ensure accuracy. All information, questions, and interactions were provided within the app based on a relative number of days before and after these events. Push notifications were used to alert patients about the newly available information actively. The timing of the push notifications was configured per information item (eg, information about hormone medication side effects was provided 3 days after the intake consultations at 11 am, and information about the preparation for the oocyte puncture appeared 2 days before the oocyte puncture at 8 pm). An overview of the content, notifications, and timing is presented in Multimedia Appendix 1.

**Figure 1:**
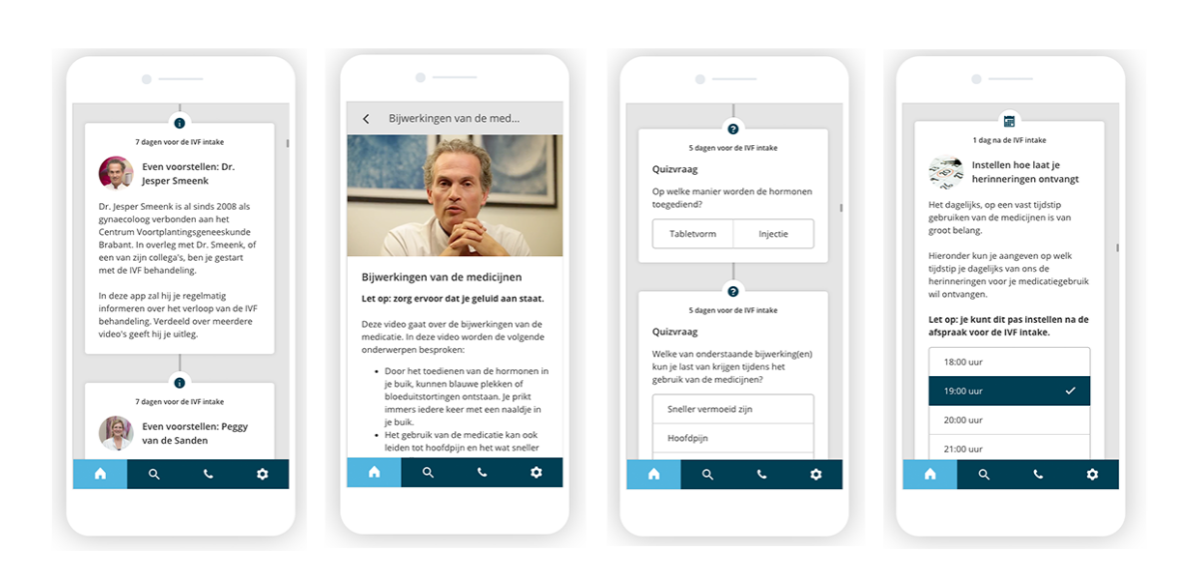
Examples of the interactive app used as an intervention in the study (in Dutch). From left to right: introduction of the different health care providers in the app, video and text information about medication usage and side effects, quiz-like questions to assess patient’s knowledge on various topics, and the configuration of patient-specific medication reminders.

The text, photos, and video used in the app were developed specifically for this trial in close collaboration with a gynecologist (JS, subspecialist reproductive medicine since 2011), a clinical embryologist (since 2004), and a specialized fertility care nurse (since 2010). Furthermore, the electronic health records of 10 patients who had previously undergone IVF or ICSI treatment were checked to determine why they had contacted the hospital. Based on this information, an interactive timeline was developed (Figure 1). All information on the timeline was presented in Dutch. No changes were made to the app’s content during the trial.

Information in the app was tailored to the ETZ hospital and based on existing protocols. Patients that used the app from IVF intake to pregnancy test after a successful embryo transfer received 52 information items and 30 push notifications. The information was disseminated over different phases of the IVF or ICSI process: introduction, welcome to the ETZ fertility center, what is IVF or ICSI, medication usage, IVF or ICSI intake consultation, medication reminders, treatment schedule (hormone injections, side effects, and echography), oocyte retrieval, embryo transfer, and a pregnancy test.

Prior to the study, 4 patients were interviewed to assess the general usefulness and usability of the app. They reported that the app would be very useful and offered no additional suggestions or changes. After the study, all the content developed for the intervention was provided to the fertility clinic, allowing them to offer it to their patients as part of the new standard of care.

### Study Outcomes

Patients’ satisfaction with the information they received during the treatment was assessed as a primary outcome. Secondary outcomes assessed patients’ level of knowledge, satisfaction with the IVF intake consultation, health care consumption, and their ability to understand the information, administer hormone injections, and manage side effects. In addition, we assessed patients’ overall satisfaction with the entire treatment process. Finally, data on app usage was continuously captured to understand better how the app is being used over time, the type of information that patients consult, and the videos they watch (Textbox 1).

**Overview of questionnaires used per outcome.**

**Outcome and questionnaire**

- **Satisfaction with the information:** A single question concerning patients’ satisfaction with the information they received during their treatment. Numeric scale rating (NRS) scores were used to measure the outcome, ranging from 0 (not satisfied at all) to 10 (extremely satisfied).
- **Level of knowledge:** The patient’s perceived level of knowledge about their cycle, administration of hormone injections, side effects of hormone injections, other medications, oocyte retrieval, embryo transfer, and pregnancy test was determined through 7 questions. All questions were scored on a 1 to 5 scale: very knowledgeable, knowledgeable, neutral, little knowledge, and very little knowledge. Sum scores ranging from 7 to 35 were used to measure the outcome.
- **General satisfaction of in vitro fertilization (IVF) intake consultation:** One question assessed patients’ overall satisfaction with the IVF intake consultation. NRS scores were used to measure the outcome, ranging from 0 (not satisfied at all) to 10 (extremely satisfied).
- **Ability to understand the information during the IVF intake consultation:** One question addressed patients’ ability to understand the information presented during the IVF intake consultation. NRS scores were used to measure the outcome, ranging from 0 (no understanding at all) to 10 (full understanding).
- **Administering hormone injections:** One question evaluated patients’ ability to administer the hormone injections at the right time. NRS score was used to measure the outcome, ranging from 0 (not capable at all) to 10 (perfectly capable).
- **Managing side-effects:** One question assessed patients’ ability to manage treatment side effects caused by the hormone injections. NRS score was used to measure the outcome, ranging from 0 (not capable at all) to 10 (perfectly capable).
- **Overall quality of the IVF treatment:** The QPP-IVF (Quality from the Patient’s Perspective of In Vitro Fertilization) questionnaire [22] assessed 3 dimensions of IVF care: medical-technical conditions (pain, physical care, and waiting time), physical-technical conditions (care room characteristics), and identity-orientated approaches (information during and after treatment, participation, responsibility or continuity, the staffs’ respect, and empathy).
- **Health care consumption:** Five questions addressed contacting the hospital in the past 7 days (in addition to planned calls or visits), medication usage, side effects, oocyte retrieval, or other topics. A 0 to 4 score was used to indicate the number of contacts.
- **App usage data:** Continuous logging of all the actions that patients perform in the app, such as opening the app, reading the information, and watching a video.

The study outcomes were measured a total of 4 times during the IVF or ICSI process (Textbox 2). The baseline measurement was taken after patients were enrolled in the study. Follow-up questionnaires were sent to both groups 2 days and 10 days after the IVF intake consultation and 5 days after the oocyte retrieval. Patients were invited to participate in the questionnaire by email. A maximum of 2 email reminders was sent if patients did not respond to the initial invite. Patients had a 7-day window to complete the questionnaires for each measurement. All outcome data were self-reported and collected using an online system. Patients who either missed the baseline measurement or more than 2 follow-up questionnaires were registered as lost to follow-up. These patients were not included in the final data analysis.

**Overview of outcomes assessed throughout the in vitro fertilization (IVF) or intracytoplasmic sperm injection (ICSI) treatment process.**

**Outcomes assessed during each stage of the IVF or ICSI**

- **Baseline:** Satisfaction with the information provided, level of knowledge, and app usage data.
- **2 days after IVF intake:** Satisfaction with the information provided, level of knowledge, general satisfaction with the IVF intake consultation, ability to understand the information during the IVF intake consultation, administering hormone injections, managing side effects, and app usage data.
- **10 days after IVF intake:** Administering hormone injections, managing side effects, app usage data, and health care consumption
- **5 days after oocyte retrieval:** Satisfaction with the information provided, level of knowledge, overall quality of IVF treatment, and app usage data

### Sample Size

The sample size calculation was based on the 2016 study, which assessed IVF patients’ experiences and satisfaction with patient information [23]. This study revealed an average satisfaction score of 7.29 (SD 2.2) on a 0 to 10 scale. In our study, we expected an average satisfaction of 8.5 (SD 1.5). We performed a power calculation on powerandsamplesize.com using 2-sided equality, α=.05, and β=.90, resulting in 33 patients in each arm. We also added a 10% dropout margin for a total of 36 patients in each arm.

### Randomization

Patients were randomized to either the control or intervention group by a computer program. Randomization was performed without block or stratification restrictions. After being allocated to one of the groups, patients received an email that included the link to the baseline questionnaire.

### Statistical Methods

For our analysis, we used an intention-to-treat approach, including all randomized patients. Normally distributed continuous variables (eg, satisfaction and level of knowledge) were presented as a mean value with the SD, and they were statistically compared between the groups using independent 2-tailed student *t*-tests. Nonnormally distributed variables were presented as a median value with the IQR. Categorical variables (eg, health care consumption) were presented as sample number and percentage and compared between groups using chi-square tests. Missing data were not replaced in any type of analysis. Patients’ level of education was divided into 2 groups for analysis: group 1 (none, elementary school, or secondary or vocational education) and group 2 (higher secondary education, pre-university education, or university education in applied sciences). *P* values of ≤.05 indicated a significant difference, and *P* values between .05 and .10 were indicated a trend. All data were analyzed using SPSS Statistics for Macintosh (version 25.0; IBM).

## Results

### Study Sample

Between June 2018 and August 2019, a total of 65 patients were willing to participate in the study of which, 2 patients got pregnant before the start of their treatment, 1 patient withdrew due to mental instability, and 8 patients dropped out due to logistical reasons. As a result, a total of 54 patients were randomized into the control and intervention groups.

**Figure 2:**
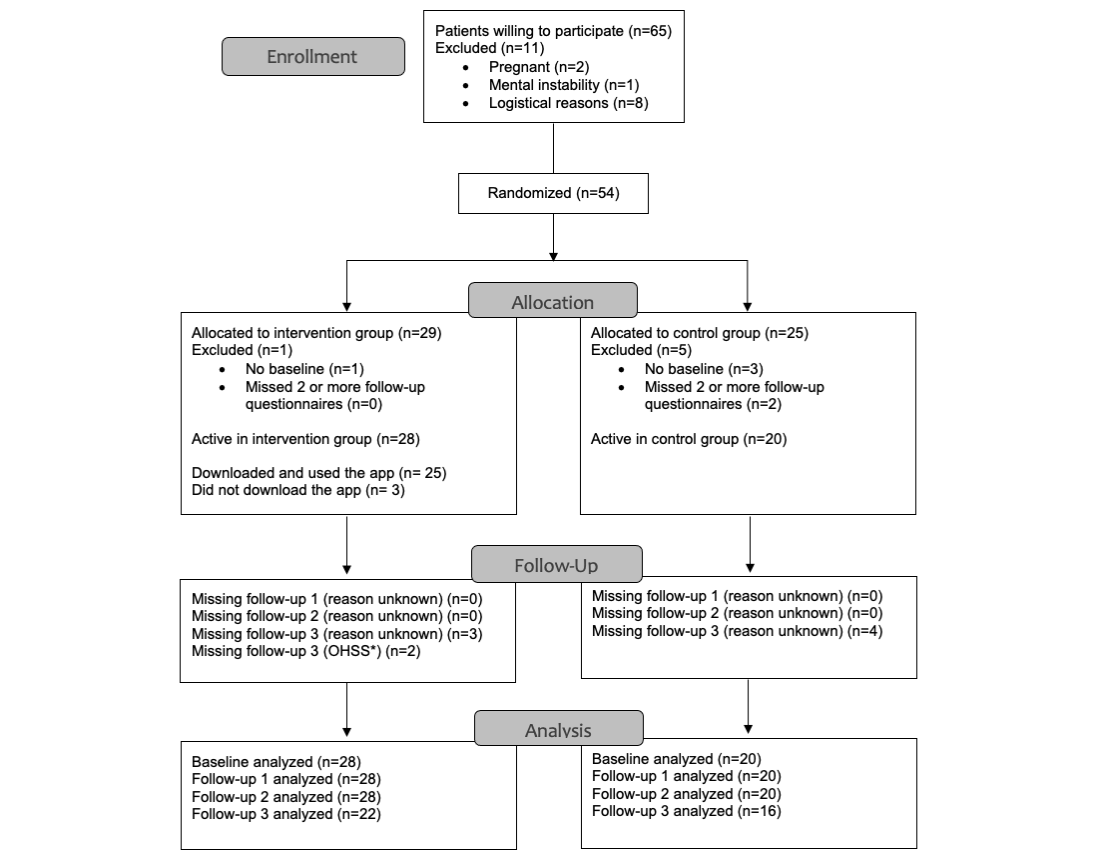
Patient flow diagram. OHSS: ovarian hyperstimulation syndrome.

Of the 54 patients in the study, 4 (7.4%) did not complete the baseline questionnaire, and 2 (3.7%) withdrew from the study for reasons unknown. In total, 28 patients were actively enrolled in the intervention group and 20 patients in the control group. In the intervention group, 25 (89.3%) participants downloaded and used the app (Figure 2). Baseline characteristics of the study population were largely similar between groups (Table 1).

**Table 1:** Patient characteristics.

Characteristics			Intervention group (n=28)		Control group (n=20)
Age (years), mean (SD)			32.39 (5.14)		32.76 (4.55)
**Education, n (%)**					
	Group 1 (low)	13 (46.4)		10 (50.0)	
	Group 2 (high)	15 (53.6)		10 (50.0)	
Years trying to get pregnant, median (IQR)			2 (1.0-3.0)		2 (1.0-3.0)
Prior IUI^a^ treatment at ETZ^b^ hospital (yes), n (%)			2 (7.14)		3 (15.0)
Treated before in another hospital (yes), n (%)			4 (14.29)		2 (10.0)

^a^IUI: intrauterine insemination.

^b^ETZ: Elisabeth-TweeSteden Ziekenhuis hospital.

### Primary Outcome

#### Patient Satisfaction With the Information Received During the Treatment

There was no difference between the 2 groups at baseline (intervention group: mean 6.56, SD 1.18 vs control group: mean 6.86, SD 1.18; *P*=.76). However, there was a significant difference in favor of the intervention group 2 days after the IVF intake consultation (intervention group: mean 8.43, SD 1.03 vs control group: mean 7.70, SD 0.66; *P*=.004). At the third and final measurement, 5 days after the oocyte retrieval, the level of satisfaction was equal between groups (intervention group: mean 8.14, SD 1.04 vs control group: mean 8.06, SD 1.44; *P*=.86; Table 2).

**Table 2:** Patient satisfaction with the information received during the treatment.

Satisfaction with information	Baseline	2 days after IVF^a^ intake	5 days after oocyte retrieval
Intervention group, mean (SD), participants	6.56 (1.18) n=28	8.43 (1.03) n=28	8.14 (1.04) n=22
Control group, mean (SD), participants	6.86 (1.18) n=20	7.70 (0.66) n=20	8.06 (1.44) n=16
*P* value	.76	.004	.86

^a^IVF: in vitro fertilization.

### Secondary Outcomes

#### Level of Knowledge

There was no difference in the level of knowledge between the 2 groups at baseline (intervention group: mean 19.00, SD 3.08 vs control group: mean 17.31, SD 3.20; *P*=.09). However, there was a significant difference in favor of the intervention group 2 days after the IVF intake consultation (intervention group: mean 27.29, SD 2.94 vs control group: mean 23.05, SD 2.76; *P*<.001). At the third and final measurement, 5 days after the oocyte retrieval, the level of knowledge was slightly higher in the intervention group, but this difference was no longer significant (intervention group: mean 27.60, SD 3.48 vs control group: mean 27.13, SD 4.01; *P*=.71; Table 3).

**Table 3:** Level of knowledge.

Level of knowledge	Baseline	2 days after IVF^a^ intake	5 days after oocyte retrieval
Intervention group, mean (SD), participants	19.00 (3.08) n=28	27.29 (2.94) n=28	27.60 (3.48) n=22
Control group, mean (SD), participants	17.31 (3.20) n=20	23.05 (2.76) n=20	27.13 (4.01) n=16
*P* value	.09	<.001	.71

^a^IVF: in vitro fertilization.

#### Satisfaction With the IVF Intake Consultation and Ability to Understand the Information

Although patients in the intervention group rated the IVF intake consultation higher than patients in the control group, there was no significant difference in their satisfaction levels (intervention group: mean 9.00, SD 8.61 vs control group: mean 8.60, SD 0.94; *P*=.13). However, patients in the intervention group reported a significantly higher score regarding their ability to understand all the information provided during the consultation (intervention group: mean 8.96, SD 1.14 vs control group: mean 7.95, SD 1.36; *P*=.01).

#### Administering Hormone Injections and Managing Side Effects

Patients’ ability to administer the hormone injections was measured 2 days after the IVF intake and showed no differences between groups (intervention group: mean 8.57, SD 1.10 vs control group: mean 8.15, SD 1.73; *P*=.31). This outcome was measured again 10 days after the IVF intake consultation with similar results (intervention group: mean 8.74, SD 1.20 vs control group: mean 8.26, SD: 1.94; *P*=.23).

Patients’ ability to manage the side effects of the hormone injections was measured 2 days after the IVF intake consultation and showed no differences between groups (intervention group: mean 7.43, SD 1.60 vs control group: mean 7.15, SD 1.47; *P*=.54). It was measured again 10 days after the IVF intake consultation with similar results (intervention group: mean 7.52, SD 1.06 vs control group: mean 7.42, SD 1.43; *P*=.79).

#### Overall Quality of the IVF Treatment

There was no difference between the groups regarding the perceived overall quality of the IVF treatment (intervention group: mean 51.65, SD 12.73 vs control group: mean 48.59, SD 9.55; *P*=.41).

#### Health Care Consumption

A trend was observed between the 2 groups concerning health care consumption. Patients in the intervention group contacted the hospital less frequently (intervention group: mean 0.44 contacts per patient, SD 0.85 vs control group: mean 0.84 contacts per patient, SD 0.69; *P*=.09).

#### App Usage Data

In total, 25 patients in the intervention group used the app 1425 times, an average of 57 times per patient. Patients primarily used a smartphone to access the information (1283/1425, 90%) compared to tablet use (142/1425, 10%). Hormone injection instructions, the side effects of medication, the first day of the IVF cycle, the oocyte retrieval, and usage of the medication capsules after the embryo transfer were consulted most frequently. During the intervention, 26 videos were offered to each patient on average. In total, these videos were viewed 618 times, an average of 24 views per patient. In addition, video-enriched information items about the start of the IVF cycle, medication side effects, oocyte retrieval, and embryo transfer were frequently viewed.

### Post-Hoc Power Analysis

Unfortunately, we could not include as many patients as we required based on the initial power calculation we performed. Therefore, to determine the strength of our results, we performed a post-hoc power calculation based on the results of our primary outcome (ie, satisfaction with the information two days after the IVF intake). It showed a power of 81%, indicating that our study was not underpowered.

## Discussion

### Principal Findings

The results of our study demonstrate the effectiveness of using an app to educate and support patients that undergo IVF treatment. Regarding the primary outcome, patients in the intervention group were more satisfied with the information they received, especially in the first stages of their treatment. Furthermore, the app positively affected patients’ knowledge about the different aspects of their IVF treatment and their ability to understand the information during the intake consultation.

To our knowledge, our study is the first to assess the effectiveness of using an app to educate IVF patients through the different stages of their treatment, offering the information promptly by using push notifications. The results on outcomes such as satisfaction and level of knowledge are in line with a 2020 review on using apps to educate patients in this timely manner [19]. Satisfactory patient information and the feeling of a (virtual) continuity of care have been indirectly associated with better individual well-being by reducing treatment concerns and enabling higher treatment tolerability [24]. The importance of information provisioning was also demonstrated by a large European study focusing on patient-centered care in fertility clinics [25]. Being more responsive to patients’ needs and expectations can lower the number of discontinued treatments because it reduces the level of emotional distress [26]. However, the results on medication adherence and the management of side effects differ from previous studies, where patients reported anxiety concerning these topics [11,27]. In our study, participants in both groups reported similar positive scores on their ability to manage their medication regimens.

### Strengths and Limitations

An important strength of our study is content development, for which we combined multiple insights from specialized fertility physicians and nurses, and embryologists. Another strength is push notifications, allowing the app to reach out to patients when new information was available actively. By delivering the most relevant information in smaller segments, patients can better process and retain the information [19,28]. The app usage statistics demonstrate patients’ willingness and need for the app, and that offering complex topics such as the start of the cycle, the oocyte retrieval, side effects of medication, and the embryo transfer through video is highly appreciated.

### Limitations

An essential limitation of the study is that we could not include as many patients as required based on the initial power calculation. It was mainly due to staffing problems at the hospital. Nevertheless, a post-hoc power analysis was performed to determine the strength of our results, demonstrating that our study was not underpowered. In addition, we did not involve patient input when deciding which content to offer through the app, including the format and timing of push notifications. This could have contributed to a more personalized experience. Finally, we used several self-reported questionnaires. Although not scientifically validated, we presented the questionnaires to patients prior to the study initiation and used the commonly applied 0 to 10 numeric rating scale mechanism to score the items.

### Clinical Implications and Future Research

All patients in the intervention group could download and use the app with no additional instructions besides those provided in the initial email, positively demonstrating the ease and acceptance of the intervention from a patient perspective. It also means that implementing such an app does not require the hospital staff to alter their routines to support patients with the app. In its current form, the app optimizes the IVF patient journey without requiring additional staff resources.

Future development and research should focus on a more personalized version of the app, including a more qualitative approach to better identify patients’ needs and new strategies to ensure the information suits their communication styles [29-31]. In addition, adjusting the information based on a patient's anxiety or depression during the different stages of the treatment can enable patients to better cope with the emotional burden of undergoing the treatment and managing related outcomes [32-34].

The app use might be extended to other phases of the IVF treatments or other treatments as well, not only to inform patients about the next step in their treatment but also to actively involve them in their treatment. For example, it would be interesting to see if the patient and health care provider’s reported quality of the first consultation would change when patients primarily use the app for educational purposes and in-person clinic visits are spent addressing questions and their priorities. In addition, using the app as a communication platform between patients and health care providers during the entire care journey could further optimize the perceived quality of care. Previous studies focusing on online IVF platforms reported positive outcomes on such ideas, but only if they are strategically implemented in the clinic as integral to the standard of care [35,36].

### Conclusions

Our study demonstrates that, in comparison with standard patient education, using an app to provide patients with timely information increases their level of satisfaction. Furthermore, using the app leads to a higher level of knowledge regarding the steps and procedures of IVF treatment. The app’s usage statistics demonstrate patients’ need for information and their willingness to use an eHealth application as part of their treatment. Future interventions might use a better patient-centered approach, for instance, collecting information about patients’ needs and expectations in preparation for their initial consultations or the treatment itself.

## Appendix

Multimedia Appendix 1 Overview and timing of the content in the app.

Multimedia Appendix 2 CONSORT-eHEALTH checklist (V 1.6.1).

## Abbreviations

CONSORT: consolidated standards for reporting trials
ETZ: Elisabeth-TweeSteden Ziekenhuis hospital
ICSI: intracytoplasmic sperm injection
IVF: in vitro fertilization
