# Supplementary material for: Supporting Women Undergoing IVF Treatment With Timely Patient Information Through an App: Randomized Controlled Trial
Source: JMIR Mhealth Uhealth. 2021 Aug 27;9(8):e28104. doi: 10.2196/28104 (PMC8433853; doi:10.2196/28104)
Supplement: Multimedia Appendix 1 [file mhealth_v9i8e28104_app1.pdf]

Appendix I: Content, Notifications and Timing

Content	Notification and timing	Time
**Welcome**		
Enter your personal code		
About the fertility clinic	7 days before first appointment	7 pm
Why it is important to be well prepared		
Introducing the team		
A short quiz		
**What is IVF treatment**		
What is IVF?	6 days before first appointment	7 pm
What are the odds?		
**Medication**	5 days before first appointment	7 pm
Different types of medications		
Commonly reported side effects		
**About the consultation**	3 days before first appointment	7 pm
Structure of the consultation		
Topics that are discussed		
What to bring with you		
**The first steps**		
How to use the medication	1 day after first appointment	11 am
Reporting the first day of your period	4 days after first appointment	11 am
When to contact the hospital	5 days after first appointment	7 pm
Ultrasound examination(s)	8 days after first appointment	7 pm
Side effects of the medication	4 days after first appointment	7 pm
**Oocyte retrieval**		
About the procedure	2 days before the oocyte retrieval	7 pm
Use the Ovitrelle medication	2 days before the oocyte retrieval	
Problems using the Ovitrelle medication	1 days before the oocyte retrieval	8 am
Practical information about the oocyte retrieval	1 days before the oocyte retrieval	7 pm
What happens during and after the procedure?	1 days before the oocyte retrieval	7 pm
Don’t forget to have breakfast and take some paracetamol	1 days before the oocyte retrieval	8 am

**Embryo transfer**		
What is an embryo transfer	2 days before the embryo transfer	7 pm
What happens after the transfer?	2 days before the embryo transfer	
Who to contact in the case of an emergency	2 days before the embryo transfer	
Meanwhile in the lab…	1 day before the embryo transfer	1 pm
Reminder: Utrogestan medication	1 day before the embryo transfer	3 pm
Practical information about the embryo transfer	1 day before the embryo transfer	7 pm
**Pregnancy test**		
When and how a pregnancy test is performed	5 days after the embryo transfer	7 pm
**Thank you**		
Discussing the outcome of the pregnancy test	10 days after the embryo transfer	7 pm
Thank you for your participation	21 days after the embryo transfer	7 pm
